# MHC class II genotype‐by‐pathogen genotype interaction for infection prevalence in a natural rodent‐*Borrelia* system

**DOI:** 10.1111/evo.14590

**Published:** 2022-08-09

**Authors:** Lars Råberg, Dagmar Clough, Åsa Hagström, Kristin Scherman, Martin Andersson, Anna Drews, Maria Strandh, Barbara Tschirren, Helena Westerdahl

**Affiliations:** ^1^ Department of Biology Lund University Lund SE‐22362 Sweden; ^2^ Centre for Ecology and Conservation University of Exeter Penryn TR10 9FE United Kingdom

**Keywords:** *Borrelia*, coevolution, frequency‐dependent selection, *Myodes glareolus*, *ospC*

## Abstract

MHC genes are extraordinarily polymorphic in most taxa. Host‐pathogen coevolution driven by negative frequency‐dependent selection (NFDS) is one of the main hypotheses for the maintenance of such immunogenetic variation. Here, we test a critical but rarely tested assumption of this hypothesis—that MHC alleles affect resistance/susceptibility to a pathogen in a strain‐specific way, that is, there is a host genotype‐by‐pathogen genotype interaction. In a field study of bank voles naturally infected with the tick‐transmitted bacterium *Borrelia afzelii*, we tested for MHC class II (*DQB*) genotype‐by‐*B. afzelii* strain interactions for infection prevalence between 10 *DQB* alleles and seven strains. One allele (DQB*37) showed an interaction, such that voles carrying DQB*37 had higher prevalence of two strains and lower prevalence of one strain than individuals without the allele. These findings were corroborated by analyses of strain composition of infections, which revealed an effect of DQB*37 in the form of lower β diversity among infections in voles carrying the allele. Taken together, these results provide rare support at the molecular genetic level for a key assumption of models of antagonistic coevolution through NFDS.

Antagonistic coevolution with pathogens, driven by negative frequency‐dependent selection (NFDS), has long been considered one of the main processes generating and maintaining genetic diversity in hosts (Haldane 1949; Woolhouse et al. 2002; Ebert and Fields 2020). However, it is often difficult to disentangle NFDS from other types of pathogen‐mediated balancing selection, such as heterozygote advantage or selection that varies in time or space due to environmental heterogeneity in pathogen abundance (Spurgin and Richardson 2010; Ebert and Fields 2020; Huang et al. 2022). As a consequence, conclusive evidence for a role of NFDS in natural host‐pathogen systems remains remarkably scarce.

One way to investigate the potential for NFDS to contribute to the maintenance of genetic variation in host‐pathogen systems is to test a key assumption specific to this process: that host alleles affect resistance/susceptibility to a pathogen in a strain‐specific way, that is, there is a host genotype‐by‐pathogen genotype interaction (H_G_×P_G_). Indeed, H_G_×P_G_ is a critical assumption of classical models of antagonistic coevolution, including gene‐for‐gene (GFG) and matching‐allele (MA) models and different variants of them (Frank 1993; Dybdahl et al. 2014; Buckingham and Ashby 2022; Fig. 1). In plant and invertebrate host‐pathogen systems, H_G_×P_G_ at the phenotypic level (i.e., in infection experiments with different clones of host and pathogen) are well documented (e.g., Carius et al. 2001; Salvaudon et al. 2007), and studies pinpointing the host loci involved are starting to emerge (e.g., Lambrechts et al. 2012; Bento et al. 2017). Thus, there is clearly scope for NFDS in many plant and invertebrate host‐pathogen systems. In contrast, in vertebrate host‐pathogen systems, evidence for H_G_×P_G_ is as yet limited.

**Figure 1:**
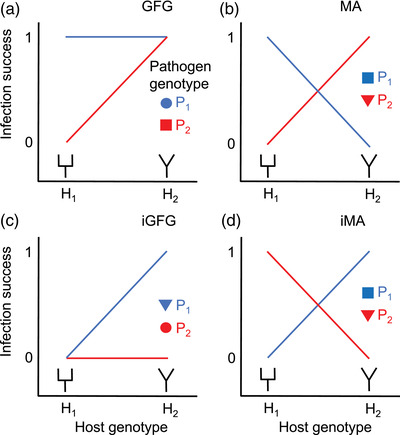
Different types of H_G_×P_G_ for resistance/susceptibility to infection as assumed in different models of host‐pathogen coevolution. (a) H_G_×P_G_ in gene‐for‐gene (GFG) models. (b) H_G_×P_G_ in matching‐allele (MA) models. (c) H_G_×P_G_ in inverse gene‐for‐gene (iGFG) models. (d) H_G_×P_G_ in inverse matching‐allele (iMA) models. Schematic shapes of host receptors and pathogen ligands under different scenarios are indicated. GFG and iMA are based on the idea of “targeted recognition,” that is, infection occurs when the pathogen can evade detection by, for example, an immune receptor (i.e., when the shape of the host receptor and pathogen ligand does not match). MA and iGFG are based on the idea of “matching infection,” that is, infection occurs when a pathogen molecule can bind to a host molecule (i.e., when the shape of the host receptor and pathogen ligand matches), for example, a host cell surface protein that mediates pathogen entry into the cell. The H_G_×P_G_ shown here represent a simplified scenario where both host and pathogen are haploid and have one locus with two alternative alleles (based on figures in Frank 1993; Fenton et al. 2009; Dybdahl et al. 2014). In the case of MHC, the situation is more complex with multiallelic diploid hosts; in our analyses, we therefore test for effects of presence/absence of each MHC allele on prevalence of pathogen strains, instead of effects of alternative alleles on prevalence as illustrated in this figure

In vertebrates, much of the research on pathogen‐mediated balancing selection has focused on MHC genes, which encode cell surface proteins playing a key role in the vertebrate immune system by presenting antigens to T lymphocytes (Radwan et al. 2020). NFDS has long been considered a potentially important driver of MHC polymorphism (Bodmer 1972; Hedrick and Thomson 1983). Yet to date, only a couple of studies have provided evidence for MHC genotype‐by‐pathogen genotype interactions, focusing on MHC class I (which encode MHC molecules expressed on all nucleated cells, presenting peptide antigens from proteins generated within the cell, including, e.g., viral proteins, to cytotoxic T cells). First, a study of virus adaptation through serial passage in inbred laboratory mouse strains congenic for different MHC class I haplotypes revealed trade‐offs in viral fitness and virulence between mouse strains, thus providing proof‐of‐concept for H_G_×P_G_ involving MHC (Kubinak et al. 2012). Second, a field study of human malaria showed that the prevalence of different *Plasmodium falciparum* genotypes differed between individuals with and without a certain MHC class I allele in a *P. falciparum* genotype‐specific way (Gilbert et al. 1998). Besides MHC, there are also a few recent human studies that have found that allelic variation at various non‐MHC genes leads to H_G_×P_G_ (Ansari et al. 2017; Lees et al. 2019; McHenry et al. 2020; Band et al. 2021). However, H_G_×P_G_ at the molecular genetic level has to the best of our knowledge never been demonstrated in a natural nonhuman vertebrate host‐pathogen system.

Here, we use a wild rodent, the bank vole (*Myodes glareolus*), and one of its natural pathogens, the tick‐transmitted bacterium *Borrelia afzelii*, to test for MHC genotype‐by‐pathogen genotype interactions. As *B. afzelii* is primarily an extracellular pathogen, we focused on MHC class II (which encodes MHC molecules expressed on professional antigen presenting cells—i.e., dendritic cells, macrophages, and B lymphocytes—presenting endocytosed antigens to T helper cells). One challenge when testing for H_G_×P_G_ is that it is difficult to know which pathogen gene might be involved. We circumvent this problem by testing for H_G_×P_G_ in the form of MHC class II genotype‐by‐*B. afzelii* strain interactions (Fig. 1), taking advantage of the fact that local populations of *B. afzelii* have a highly clonal genetic structure so that different strains can be distinguished by genotyping a single locus (here *ospC*) (Hellgren et al. 2011).

## Methods

### STUDY SYSTEM

The bank vole is a small, sexually monomorphic rodent and one of the most common and widely distributed mammals in Europe (Wilson et al. 2017). At the study site, bank voles mainly reproduce from May to September. Based on recapture data, individuals born early in the reproductive season reproduce the same season and rarely survive to the following year, whereas individuals born late defer reproduction to the following season (L. Råberg, unpubl. data).

The MHC class II molecule is a dimer composed of an α and β chain. Most mammals have three loci with classical MHC II genes (*DQ*, *DR*, and *DP*; although in, for example, *Mus musculus*, *DP* genes are pseudogenes), with each locus containing one or more gene copies encoding α chains (*DQA*, etc.) and one or more gene copies encoding β chains (*DQB*, etc.) (Kumánovics et al. 2003). Genes encoding β chains are generally more polymorphic than genes encoding α chains (Murphy and Weaver 2017). Moreover, previous analyses of bank vole MHC class II showed a larger number of positively selected sites in *DQB* than *DRB* genes (Scherman et al. 2014). In the present study, we therefore focused on *DQB*. Bank voles in the present study population can have up to eight *DQB* alleles, hence at least four *DQB* gene copies (Scherman et al. 2014).

*Borrelia afzelii* is a tick‐transmitted bacterium and one of the causative agents of Lyme disease in humans (Kurtenbach et al. 2006). The natural hosts of *B. afzelii* are mainly rodents and shrews (Kurtenbach et al. 2002; Hellgren et al. 2011). At our study site, the bank vole is the most abundant host species (Råberg et al. 2017). *Borrelia afzelii* infections in bank voles are often chronic (Gern et al. 1994), but some individuals apparently clear infection, as indicated by loss of infection over winter (Scherman 2015). The overall prevalence of *B. afzelii* in bank voles at Kalvs mosse during May‐October is 23.4% (Råberg et al. 2017). Prevalence increases with age (as determined by body mass), from a few percent in juveniles (<15 g) to around 30% in adults (≥20 g) (Andersson et al. 2013; Tschirren et al. 2013), presumably because older individuals have had longer time to acquire infection. Local populations of *B. afzelii* have a highly clonal genetic structure with virtually perfect associations between alleles at different loci, also between chromosomal and plasmid loci (Hellgren et al. 2011). Thus, different strains of *B. afzelii* can be distinguished by genotyping a single locus. We distinguished different strains by sequence genotyping of *ospC*, a plasmid‐borne single‐copy gene that is the most polymorphic locus in the *Borrelia* genome (Haven et al. 2011). *ospC* encodes the outer surface protein C (OspC), which induces a protective antibody response in the host (Jacquet et al. 2015). Thus, *ospC* could potentially be the focal gene involved in an H_G_×P_G_ with *DQB*, but a *DQB* × *ospC* could equally well be due to another *B. afzelii* gene encoding an antigen that is in linkage disequilibrium with *ospC*, and we stress that we here use *ospC* as a marker of *B. afzelii* strain identity. A given community of *B. afzelii* hosts (e.g., in a small forest) harbors five to 19 *ospC* strains (Hellgren et al. 2011; Durand et al. 2017). There are no differences in strain frequencies between host species (Råberg et al. 2017) and strain frequencies at a given site are stable over time for at least a decade (Durand et al. 2017; Råberg et al. 2017). Individual hosts are often infected with more than one *ospC* strain simultaneously (Strandh and Råberg 2015).

### FIELD SAMPLING

Animals for this study were trapped at Kalvs mosse, a damp deciduous wood (ca. 24 ha) at Revingehed in southern Sweden (55°42ʹN, 13°29ʹE), in May‐October 2006–2014. Animals were trapped with live traps (Ugglan special, GrahnAB, Sweden) baited with grains and apple or carrot. From each individual, we took a skin biopsy (∅ 2 mm) from the ear for DNA extraction. We also recorded body mass to the nearest 0.1 g using a Pesola spring balance. During 2007–2008, we collected longitudinal data by trapping bank voles about every 6 weeks during May‐October and tagging each individual with a transponder (Trovan ID‐100 Unique) subcutaneously implanted on the back, which allowed identification of each individual upon recapture.

### DQB AND ospC SEQUENCING

Skin biopsies were stored in 70% ethanol until DNA extraction using the protocol of Laird et al. (1991).

*Borrelia afzelii‐*infected bank voles were identified by real‐time PCR of *flaB* (Råberg 2012). To determine which *ospC* strains an infected vole carried, we used 454 amplicon pyrosequencing, as described in Strandh and Råberg (2015). The *ospC* dataset used in the present study is the same as in Råberg et al. (2017).

*Borrelia afzelii*‐infected voles were genotyped at *DQB* by amplifying 205 out of 272 bp in exon 2 using the primers MyglDQBfw and MyglDQBrv (Scherman et al. 2014), followed by amplicon sequencing. Exon 2 contains the majority of the peptide binding residues and is the most polymorphic exon in this gene (Scherman et al. 2014). Two different sequencing methods were used: 300 bp paired‐end Illumina MiSeq sequencing (*N* = 265 in the final dataset) and 454 pyrosequencing (*N* = 36 in the final dataset; a subset of the data from Scherman et al. 2021). Libraries were prepared and the data filtered as described in Scherman et al. (2021). The concordance between Illumina MiSeq sequencing and 454 pyrosequencing of MHC is high (Razali et al. 2017). The reproducibility (number of *DQB* alleles detected in all replicates from an individual/total number of *DQB* alleles detected in that individual) was in all cases 100% for both Illumina MiSeq sequencing (samples from 34 longitudinally sampled voles; 2–4 samples/vole) and 454 pyrosequencing (13 technical duplicates; Scherman et al. 2021).

### STATISTICAL ANALYSES

Analyses of repeatability of infection status with each *ospC* strain in infected bank voles were performed with rptR (Stoffel et al. 2017) in R 4.1.0 (RCoreTeam 2021), using rptBinary (separate analysis for each *ospC* strain). Link‐scale approximation repeatabilities are reported.

Longitudinal analyses of number of *ospC* strains in infected bank voles were performed with proc mixed in SAS 9.4 (SAS Institute) with individual as random effect. Number of *ospC* strains was square‐root transformed. We used general linear models with square‐root‐transformed data rather generalized linear models with Poisson distribution for the analysis of number of strains because the data were severely underdispersed.

Tests for effects of *DQB* × *ospC* on infection prevalence were performed as Generalized Linear Mixed Models (GLMM) with proc glimmix in SAS 9.4, with binomial error distribution. In all glimmix analyses, we used Laplace approximation, and assessed statistical significance of fixed and random effects by *χ* ^2^ and likelihood ratio (LR) tests, respectively, as recommended by Bolker et al. (2009). To examine the effects underlying a *DQB* × *ospC* interaction, we used the “slice” option in proc glimmix to test for effects of presence/absence of a particular *DQB* allele on prevalence of each *ospC* strain separately.

Analyses of effects of *DQB* genotype on *ospC* strain composition of infections were performed by PERMANOVA (Anderson 2001) and PERMDISP (Anderson 2006; Anderson et al. 2006), using the functions adonis2 and betadisper, respectively, in the vegan R package (Oksanen et al. 2020). PERMANOVA primarily tests for differences between groups (here individuals with and without a specific *DQB* allele) in location in multivariate space, whereas PERMDISP tests for differences in dispersion (i.e., β diversity) between groups. Note, however, that results from PERMANOVA may be confounded by differences in dispersion between groups (Warton et al. 2012; Anderson and Walsh 2013). For all analyses of *B. afzelii* community composition, we used Euclidean distances based on presence/absence of *ospC* strains. We chose Euclidean distances, rather than, for example, Bray‐Curtis distances that are more commonly used in analyses of microbial communities, because Euclidean distances take joint absences into account (Anderson et al. 2011). As we are interested in effects of host genotype on resistance/susceptibility to infection with particular *ospC* strains, joint absences are thus informative. To check the robustness of the results to the choice of distance metric, we also performed analyses based on Bray‐Curtis (i.e., Sörensen, when analyzing presence/absence as done here) distances.

Date was coded as days since 1st January. Continuous variables (body mass and date) were *Z* transformed. Figures were generated with ggplot2 (Wickham 2016) and corrplot (Wei and Simko 2021).

## Results

Data on *ospC* strain infection status were available from 395 *B. afzelii‐*positive samples from bank voles collected during May‐Oct 2006–2014 (same dataset as in Råberg et al. 2017). Fifty‐six of these were recaptures, meaning we had *ospC* data from 339 individual bank voles. From 301 of these, we obtained data on *DQB* genotype; these 301 represent the dataset used for all analyses below (except longitudinal analyses).

### BORRELIA INFECTIONS

Bank voles were infected with 11 different *B. afzelii ospC* strains. Four of these occurred in ≤2 bank voles each and were excluded from further analyses. As in previous analyses covering all host species (Råberg et al. 2017), the frequencies of the seven common *ospC* strains in bank voles did not vary among years (Fisher's exact test: *P* = 0.81). The number of *ospC* strains in an infected host individual ranged between one and seven (Fig. S1).

To test if infection status with each *ospC* strain was consistent over time, we used longitudinal data from recaptured voles (98 samples from 42 individuals; two samples from 30 individuals, three samples from 10 individuals, and four samples from 2 individuals; in all cases, individuals were resampled within a calendar year). For each of the seven common *ospC* strains, we estimated the repeatability of infection status. The repeatability (link‐scale approximation) ranged from 0.29 (*P* = 0.02) for *ospC2* to 0.94 (*P* < 0.001) for *ospC1*, showing that individuals were consistently infected or uninfected with each of the seven strains during a season.

To investigate how the number of strains in infected individuals varied over time, we performed a general linear mixed model with number of *ospC* strains (square‐root transformed) against year, month (fixed factors), number of days after first sample of a given individual (covariate), and individual (random effect). The number of *ospC* strains varied among individuals (LR: *χ* ^2^ = 43.0, *P* < 0.0001) and increased over time (*F*_1,55_ = 7.23, *P* = 0.009), but there were no effects of month, year, or their interaction (all *P* ≥ 0.11; Fig. S2). As the number of strains in an individual increased slightly over time, we used the last sample from recaptured individuals in the cross‐sectional analyses below.

### BANK VOLE DQB

The proportion of bank voles carrying each of the 48 *DQB* alleles observed in the study population is shown in Figure S3a. We selected alleles that occurred in ≥10% of bank voles for further analyses.

Pairwise correlations among all alleles that occurred in ≥10% of bank voles are shown in Figure S3b. In case alleles were strongly associated (*r* ≥ 0.8), we selected the allele with highest frequency for further analyses. This filtering left us with 10 alleles: MyglDQB*01, *02, *06, *09, *11, *12, *13, *16, *37, and *73.

### DQB × ospC

To test for *ospC* strain‐specific effects of *DQB* alleles, we first performed GLMMs with infection status of a given *ospC* (coded as 0/1) against *ospC* strain (fixed effect, seven levels), presence/absence of a *DQB* allele (fixed effect), and *ospC* strain × *DQB* allele. To account for the nonindependence of infections with each *ospC* strain within each individual vole, we also included the factor individual as a random effect. We performed separate analyses for each of the 10 *DQB* alleles.

**Figure 2:**
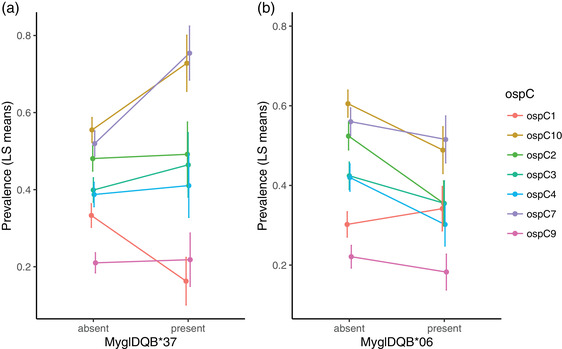
Effects of *DQB* alleles on prevalence of different *ospC* strains in bank voles. Prevalence estimated as LS means ± SE from models in Tables S1 and S2. (a) Prevalence of each *ospC* strain in bank voles with and without MyglDQB*37. (b) Prevalence of each *ospC* strain in bank voles with and without MyglDQB*06

In case of MyglDQB*37, there was a significant MyglDQB*37 × *ospC* interaction (*P* = 0.040; see Table S1 for full model details; Fig. 2a). In case of MyglDQB*06, there was a significant main effect (*P* = 0.0054; see Table S2 for full model details; Fig. 2b). For all other alleles, both the main effect and interaction were nonsignificant (*P* ≥ 0.09 and *P* ≥ 0.22, respectively). The effects of MyglDQB*37 × *ospC* and MyglDQB*06 remained significant when including both terms in the same model (*P* = 0.041 and *P* = 0.0054, respectively). Examination of the MyglDQB*37 × *ospC* interaction showed that MyglDQB*37 had a positive effect on the prevalence of *ospC7* and *ospC10* (*P* = 0.0086 and *P* = 0.048, respectively), a negative effect on *ospC1* prevalence (*P* = 0.045), but no effect on the prevalence of the remaining four *ospC* strains (*P* ≥ 0.46).

To identify potentially confounding factors, we tested for effects of body mass (as a proxy for age), date, and their quadratic terms and interaction on prevalence of *ospC* strains. Prevalence increased with body mass (body mass: *χ* ^2^ = 15.14, df = 1, *P* < 0.0001; body mass^2^: *χ* ^2^ = 5.32, df = 1, *P* = 0.026), but there were no effects of date (*P* = 0.73), date^2^ (*P* = 0.21), or body mass × date (*P* = 0.76). The effects of MyglDQB*37 × *ospC* and MyglDQB*06 were, however, not affected by inclusion of body mass and body mass^2^ in the models (MyglDQB*37 × *ospC*: *P* = 0.04; MyglDQB*06: *P* = 0.0061).

### ospC STRAIN COMPOSITION

The conventional tests for *DQB* × *ospC* presented above were complemented with tests of effects of *DQB* alleles on multivariate *ospC* strain composition. Specifically, we analyzed two aspects of strain composition: mean abundance and variability (i.e., β diversity), corresponding to “location” and “dispersion” in multivariate space, respectively (Warton et al. 2012).

**Figure 3:**
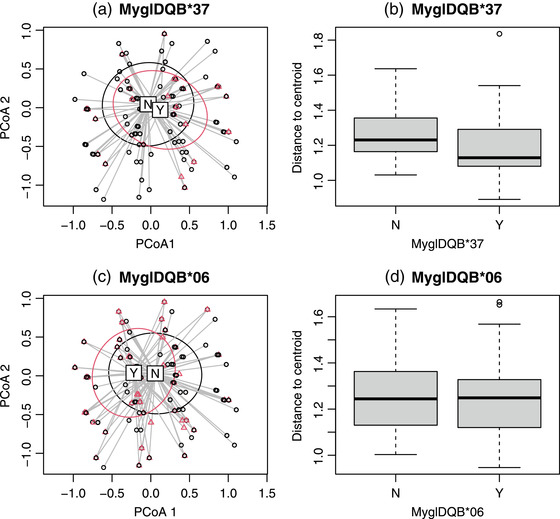
Effects of *DQB* alleles on *ospC* strain composition. (a) PCoA plot based on Euclidean distances where red points are voles with allele DQB*37 and black points are voles without DQB*37. Ellipses are SD. “N” and “Y” indicate centroid for voles with and without DQB*37, respectively. (b) Box plot of distance to centroid (as a measure of “dispersion”) for voles without (“N”) and with (“Y”) DQB*37. (c) PCoA plot based on Euclidean distances where red points are voles with DQB*06 and black points are voles without DQB*06. Ellipses are SD. “N” and “Y” indicate centroid for voles with and without DQB*06, respectively. (d) Box plot of distance to centroid for voles without (“N”) and with (“Y”) allele DQB*06. Note that in (a) and (c) there are many overlapping points, because many voles had the same composition of *ospC* strains

To test for effects of *DQB* alleles on abundance of specific *ospC* strains, we used PERMANOVA and performed separate tests with presence/absence of each of the 10 *DQB* alleles as factor. When using Euclidean distances, there were significant effects of MyglDQB*37 (*F*_1,299_ = 2.22, *P* = 0.033; Fig. 3a) and MyglDQB*06 (*F*_1,299_ = 2.22, *P* = 0.034; Fig. 3c; for all other alleles, *P* ≥ 0.30). When including both MyglDQB*37 and *06 in the same model, both remained significant (regardless of which order they were entered). When using Bray‐Curtis distances, only the effect of MyglDQB*37 was significant (*F*_1,299_ = 3.15, *P* = 0.024; all other *P* ≥ 0.26).

To test for effects of *DQB* alleles on variability of *ospC* strain composition, we used PERMDISP and performed separate tests with presence/absence of each *DQB* allele as factor. When using Euclidean distances, there was a significant effect of MyglDQB*37 (*F*_1,299_ = 8.48, *P* = 0.0039; all other *P* ≥ 0.18; Fig. 3b,d). When using Bray‐Curtis distances, there were effects of both MyglDQB*37 and MyglDQB*06 (*P* = 0.0090 and *P* = 0.0079, respectively).

## Discussion

Here, we report the first direct test for MHC class II genotype‐by‐pathogen genotype interaction (H_G_×P_G_), a critical assumption behind the idea that polymorphism at MHC is a result of host‐pathogen coevolution driven by NFDS. Using two different analytical approaches, we found evidence that allele MyglDQB*37 has strain‐specific effects on *B. afzelii* prevalence.

We acknowledge that at *P* = 0.04, the MyglDQB*37 × *ospC* interaction does not reach significance when correcting for the number of genetic comparisons performed (a Bonferroni‐corrected α‐value would be 0.05/10). However, an effect of MyglDQB*37 was also found in the analysis of strain composition (PERMDISP, at *P* = 0.0039). Moreover, a previous study that compared *B. afzelii‐*infected and uninfected bank voles (but without determining *ospC* composition of infections) showed that MyglDQB*37 is associated with *B. afzelii* infection status. Specifically, voles carrying a haplotype including MyglDQB*37 (the only haplotype with that allele) had higher prevalence of infection (Scherman et al. 2021), an observation that fits with the results of the present study where infected voles carrying MyglDQB*37 had higher prevalence of the two most abundant *ospC* strains. Taken together, this indicates that the MyglDQB*37 × *ospC* interaction observed in the present study represents a true biological pattern, rather than a type I error.

There are two basic types of models of antagonistic coevolution by NFDS: MA models and GFG models (Frank 1993; Agrawal and Lively 2002; Dybdahl et al. 2014). MA type models (including inverse MA) assume H_G_×P_G_ where host genotypes have opposite effects on resistance to different pathogen genotypes. GFG type models (including inverse GFG) instead assume that host genotypes differ in the range of pathogen genotypes they are resistant (or susceptible) to, such that one host genotype is resistant (or susceptible) to one or a few pathogen genotypes, whereas another host genotype is universally resistant (or susceptible) to all pathogen genotypes (Fig. 1). It is important to distinguish between these two types of models because MA models readily generate balanced polymorphisms, as resistance to one pathogen genotype comes with a cost in the form of susceptibility to another pathogen genotype. In contrast, the GFG scenario requires that resistance carries a cost in another currency to maintain polymorphism (Agrawal and Lively 2002).

Examination of the MyglDQB*37 × *ospC* interaction showed it has elements of both MA and GFG type H_G_×P_G_. Voles with MyglDQB*37 had higher prevalence of *ospC7* and *ospC10* and lower prevalence of *ospC1* than voles without MyglDQB*37. Hence, MyglDQB*37 appears to have opposite effects on resistance to *ospC7/ospC10* and *ospC1*, consistent with the MA/inverse MA scenario (note that the lines for *ospC7/ospC10* vs. *ospC1* do not cross each other—like in the schematic representation of the MA/inverse MA scenarios in Fig. 1b,d—because *ospC* strains differ in abundance in the tick population, so that exposure rates differs; Råberg et al. 2017). This type of H_G_×P_G_ can maintain polymorphism on its own, as resistance to *ospC1* comes at a cost in the form of susceptibility to *ospC7* and *ospC10*. In contrast, a comparison of *ospC1* or *ospC7/ospC10* against the other four *ospC* strains (*ospC2*, *ospC3*, *ospC4*, and *ospC9*) reveals a pattern consistent with GFG/inverse GFG. For example, MyglDQB*37 increased resistance to *ospC1* but had no effect on resistance to *ospC2*, *ospC3*, *ospC4*, and *ospC9*. For this type of H_G_×P_G_ to maintain polymorphism, there needs to be a cost of the resistance allele in another currency that reduces the fitness of the resistance allele in the absence of infection (Agrawal and Lively 2002). In case of MHC, one potential type of cost is autoimmunity. Indeed, in humans, polymorphisms in the MHC region, in particular in MHC II genes, are associated with a number of autoimmune diseases (Karnes et al. 2017). The significance of autoimmunity in wild vertebrates, in particular short‐lived ones like the bank vole, is virtually unknown, however.

To our knowledge, the only previous field study of a natural host‐pathogen system that has explicitly tested for MHC genotype‐by‐pathogen genotype interactions is the study of human *P. falciparum* infection by Gilbert et al. (1998). The MHC class II genotype‐by‐*B. afzelii* genotype interaction observed in the present study is similar to the MHC class I genotype‐by‐*P. falciparum* genotype interaction found by Gilbert et al. (1998) in at least two ways. First, in both cases only one MHC allele was involved in an H_G_×P_G_ (one of 10 tested MHC class II alleles in the present study; one of 12 tested MHC class I and II alleles/haplotypes in Gilbert et al. 1998). Second, the strength of the effect of the interaction term is similar (*P* = 0.04 with *N* = 301 in the present study; *P* = 0.012 with *N* = 1207 in Gilbert et al. 1998) and probably in the range that can be expected for field studies that contain considerable noise due to chance exposure to different pathogen genotypes and other factors.

Our direct test for H_G_×P_G_ using GLMMs was complemented with analyses of effects of specific *DQB* alleles on *ospC* strain composition. These analyses corroborated our conclusion that MyglDQB*37 has strain‐specific effects on prevalence. The effect of MyglDQB*37 on strain composition appears to be primarily driven by a difference in dispersion (β diversity) between voles with and without MyglDQB*37, with strain composition of infections in voles carrying this allele being more homogenous than infections in voles without this allele. In contrast, allele MyglDQB*06, which had an overall, rather than strain‐specific, effect on infection prevalence in the GLMM, affected the multidimensional location, but not dispersion, of *B. afzelii* strain composition. Analyses of strain composition of infection, as used here, may be particularly valuable in cases where there are a large number of pathogen strains, and can complement conventional tests for H_G_×P_G_ when quantifying strain‐specific resistance.

To conclude, our study is the first field study to provide evidence for H_G_×P_G_ in a nonhuman vertebrate host‐pathogen system and shows that antagonistic coevolution driven by NFDS may contribute to the maintenance of the extraordinary polymorphism of MHC class II genes. The MHC genotype‐by‐*ospC* strain interaction observed in our study has elements of both GFG and MA type H_G_×P_G_. Further analyses of other vertebrate host‐pathogen system would be desirable to investigate how common H_G_×P_G_ are and whether they generally are of GFG or MA type.

## AUTHOR CONTRIBUTIONS

LR, KS, and HW conceived the study. LR, KS, MA, and BT performed field work and general laboratory work. DC, KS, and AD generated *DQB* data. ÅH and MS generated *ospC* data. LR performed data analysis. LR wrote the first version of the manuscript and BT and HW contributed substantially to revisions. All authors approved the final version.

## CONFLICT OF INTEREST

The authors declare no conflict of interest.

## DATA ARCHIVING

Data have been deposited at SRA (PRJNA276063 and PRJNA373990) and Dryad (https://doi.org/10.5061/dryad.fttdz08w9).

Associate Editor: G.D.Hurst

Handling Editor: T.Chapman

## Supporting information

Fig S1. Frequency distribution of number of ospC strains per infected vole.
Fig S2. Longitudinal data on number of ospC strains of infected voles. *N* = 48 voles, with two to four data points per vole. Lines are slightly “dodged” both x‐wise and y‐wise to reduce overlap.
Fig S3. a) Proportion of bank voles carrying each DQB allele observed in the study population. Note that proportions given here are conservative estimates of allele frequencies in the population, as some individuals may be homozygous. b) Correlations among DQB alleles that occurred in at least 10% of bank voles. Alleles are ordered according to their frequency (decreasing from left to right).

## References

[evo14590-bib-0001] Agrawal, A. & Lively, C.M. (2002) Infection genetics: gene‐for‐gene versus matching‐ alleles models and all points in between. Evol. Ecol. Res., 4, 79–90.

[evo14590-bib-0002] Anderson, M.J. (2001) A new method for non‐parametric multivariate analysis of variance. Austral. Ecol., 26, 32–46.

[evo14590-bib-0003] ———. (2006) Distance‐based tests for homogeneity of multivariate dispersions. Biometrics, 62, 245–253. 16542252 10.1111/j.1541-0420.2005.00440.x

[evo14590-bib-0004] Anderson, M.J. & Walsh, D.C. (2013) PERMANOVA, ANOSIM, and the Mantel test in the face of heterogeneous dispersions: what null hypothesis are you testing? Ecol. Monogr., 83, 557–574.

[evo14590-bib-0005] Anderson, M.J. , Crist, T.O. , Chase, J.M. , Vellend, M. , Inouye, B.D. , Freestone, A.L. , Sanders, N.J. , Cornell, H.V. , Comita, L.S. , Davies, K.F. , et al. (2011) Navigating the multiple meanings of β diversity: a roadmap for the practicing ecologist. Ecol. Lett., 14, 19–28. 21070562 10.1111/j.1461-0248.2010.01552.x

[evo14590-bib-0006] Anderson, M.J. , Ellingsen, K.E. & McArdle, B.H. (2006) Multivariate dispersion as a measure of beta diversity. Ecol. Lett., 9, 683–693. 16706913 10.1111/j.1461-0248.2006.00926.x

[evo14590-bib-0007] Andersson, M. , Scherman, K. & Råberg, L. (2013) Multiple‐strain infections of *Borrelia afzelii*: a role for within‐host interactions in the maintenance of antigenic diversity? Am. Nat., 181, 545–554. 23535618 10.1086/669905

[evo14590-bib-0008] Ansari, M.A. , Pedergnana, V. , Ip, C.L.C. , Magri, A. , Von Delft, A. , Bonsall, D. , *et al.* (2017) Genome‐to‐genome analysis highlights the effect of the human innate and adaptive immune systems on the hepatitis C virus. Nat. Genet., 49, 666–673. 28394351 10.1038/ng.3835 PMC5873514

[evo14590-bib-0009] Band, G. , Leffler, E.M. , Jallow, M. , Sisay‐Joof, F. , Ndila, C.M. , Macharia, A.W. , *et al.* (2021) Malaria protection due to sickle haemoglobin depends on parasite genotype. Nature, 602, 106–111. 34883497 10.1038/s41586-021-04288-3 PMC8810385

[evo14590-bib-0010] Bento, G. , Routtu, J. , Fields, P.D. , Bourgeois, Y. , Du Pasquier, L. & Ebert, D. (2017) The genetic basis of resistance and matching‐allele interactions of a host‐parasite system: the *Daphnia magna*‐*Pasteuria ramosa* model. PLoS Genet., 13, e1006596. 28222092 10.1371/journal.pgen.1006596 PMC5340410

[evo14590-bib-0011] Bodmer, W.F. (1972) Evolutionary significance of the HL‐A system. Nature, 237, 139–183. 4113158 10.1038/237139a0

[evo14590-bib-0012] Bolker, B.M. , Brooks, M.E. , Clark, C.J. , Geange, S.W. , Poulsen, J.R. , Stevens, M.H.H. , *et al.* (2009) Generalized linear mixed models: a practical guide for ecology and evolution. Trends Ecol. Evol., 24, 127–135. 19185386 10.1016/j.tree.2008.10.008

[evo14590-bib-0013] Buckingham, L.J. & Ashby, B. (2022) Coevolutionary theory of hosts and parasites. J. Evol. Biol., 35, 205–224. 35030276 10.1111/jeb.13981 PMC9305583

[evo14590-bib-0014] Carius, H.J. , Little, T.J. & Ebert, D. (2001) Genetic variation in a host‐parasite association: potential for coevolution and frequency‐dependent selection. Evolution, 55, 1136–1145. 11475049 10.1111/j.0014-3820.2001.tb00633.x

[evo14590-bib-0015] Durand, J. , Jacquet, M. , Rais, O. , Gern, L. & Voordouw, M.J. (2017) Fitness estimates from experimental infections predict the long‐term strain structure of a vector‐borne pathogen in the field. Sci. Rep, 7, 1851. 28500292 10.1038/s41598-017-01821-1 PMC5431797

[evo14590-bib-0016] Dybdahl, M.F. , Jenkins, C.E. & Nuismer, S.L. (2014) Identifying the molecular basis of host‐parasite coevolution: merging models and mechanisms. Am. Nat., 184, 1–13. 24921596 10.1086/676591

[evo14590-bib-0017] Ebert, D. & Fields, P.D. (2020) Host–parasite co‐evolution and its genomic signature. Nat. Rev. Genet., 21, 754–768. 32860017 10.1038/s41576-020-0269-1

[evo14590-bib-0018] Fenton, A. , Antonovics, J. & Brockhurst, M.a. (2009) Inverse‐gene‐for‐gene infection genetics and coevolutionary dynamics. Am. Nat, 174, E230‐42. 19852618 10.1086/645087

[evo14590-bib-0019] Frank, S.A. (1993) Specificity versus detectable polymorphism in host‐parasite genetics. Proc. R. Soc. London. B, 254, 191–197. 10.1098/rspb.1993.0145 8108452

[evo14590-bib-0020] Gern, L. , Siegenthaler, M. , Hu, C.M. , Humair, P.F. & Moret, J. (1994) Borrelia burgdorferi in rodents (*Apodemus flavicollis* and *A. sylvaticus*): duration and enhancement of infectivity for Ixodes ricinus ticks. Eur. J. Epidemiol., 10, 75–80. 7957795 10.1007/BF01717456

[evo14590-bib-0021] Gilbert, S.C. , Plebanski, M. , Gupta, S. , Morris, J. , Cox, M. , Aidoo, M. , *et al.* (1998) Association of malaria parasite population structure, HLA, and immunological antagonism. Science, 279, 1173–1177. 9469800 10.1126/science.279.5354.1173

[evo14590-bib-0022] Haldane, J.B.S. (1949) Disease and evolution. La Ricerca Sci., 19, 68–76.

[evo14590-bib-0023] Haven, J. , Vargas, L.C. , Mongodin, E.F. , Xue, V. , Hernandez, Y. , Pagan, P. , *et al.* (2011) Pervasive recombination and sympatric genome diversification driven by frequency‐dependent selection in *Borrelia burgdorferi*, the Lyme disease bacterium. Genetics, 189, 951–966. 21890743 10.1534/genetics.111.130773 PMC3213364

[evo14590-bib-0024] Hedrick, P.W. & Thomson, G. (1983) Evidence for balancing selection at HLA. Genetics, 104, 449–456. 6884768 10.1093/genetics/104.3.449 PMC1202087

[evo14590-bib-0025] Hellgren, O. , Andersson, M. & Råberg, L. (2011) The genetic structure of *Borrelia afzelii* varies with geographic but not ecological sampling scale. J. Evol. Biol., 24, 159–167. 20964784 10.1111/j.1420-9101.2010.02148.x

[evo14590-bib-0026] Huang, W. , Dicks, K.L. , Hadfield, J.D. , Johnston, S.E. , Ballingall, K.T. & Pemberton, J.M. (2022) Contemporary selection on MHC genes in a free‐living ruminant population. Ecol. Lett., 25, 828–838. 35050541 10.1111/ele.13957 PMC9306867

[evo14590-bib-0027] Jacquet, M. , Durand, J. , Rais, O. & Voordouw, M.J. (2015) Cross‐reactive acquired immunity influences transmission success of the Lyme disease pathogen, *Borrelia afzelii* . Infect. Genet. Evol., 36, 131–140. 26384476 10.1016/j.meegid.2015.09.012

[evo14590-bib-0028] Karnes, J.H. , Bastarache, L. , Shaffer, C.M. , Gaudieri, S. , Xu, Y. , Glazer, A.M. , *et al.* (2017) Phenome‐wide scanning identifies multiple diseases and disease severity phenotypes associated with HLA variants. Sci. Transl. Med., 9, eaai8708. 28490672 10.1126/scitranslmed.aai8708 PMC5563969

[evo14590-bib-0029] Kubinak, J.L. , Ruff, J.S. , Whitney, C. , Slev, P.R. & Potts, W.K. (2012) Experimental viral evolution to specific host MHC genotypes reveals fitness and virulence trade‐offs in alternative MHC types. Proc. Natl. Acad. Sci. U. S. A, 109, 3422–3427. 22323587 10.1073/pnas.1112633109 PMC3295311

[evo14590-bib-0030] Kumánovics, A. , Takada, T. & Fischer Lindahl, K. (2003) Genomic organization of the mammalian MHC. Ann. Rev. Immunol., 21, 629–657. 12500978 10.1146/annurev.immunol.21.090501.080116

[evo14590-bib-0031] Kurtenbach, K. , De Michelis, S. , Etti, S. , Schäfer, S.M. , Sewell, H.‐S. , Brade, V. , *et al.* (2002) Host association of *Borrelia burgdorferi* sensu lato–the key role of host complement. Trends Microbiol., 10, 74–79. 11827808 10.1016/s0966-842x(01)02298-3

[evo14590-bib-0032] Kurtenbach, K. , Hanincová, K. , Tsao, J.I. , Margos, G. , Fish, D. & Ogden, N.H. (2006) Fundamental processes in the evolutionary ecology of *Lyme borreliosis* . Nat. Rev. Microbiol., 4, 660–669. 16894341 10.1038/nrmicro1475

[evo14590-bib-0033] Laird, P. , Zijderveld, A. , Linders, K. & Rudnicki, M. (1991) Simplified mammalian DNA isolation procedure. Nucleic Acids Res., 19, 4293. 1870982 10.1093/nar/19.15.4293 PMC328579

[evo14590-bib-0034] Lambrechts, L. , Quillery, E. , Noel, V. , Richardson, J.H. , Jarman, R.G. , Scott, T.W. , *et al.* (2012) Specificity of resistance to dengue virus isolates is associated with genotypes of the mosquito antiviral gene Dicer‐2. Proc. R. Soc. B Biol. Sci, 280, 20122437–20122437. 10.1098/rspb.2012.2437 PMC3574411 23193131

[evo14590-bib-0035] Lees, J.A. , Ferwerda, B. , Kremer, P.H.C. , Wheeler, N.E. , Serón, M.V. , Croucher, N.J. , *et al.* (2019) Joint sequencing of human and pathogen genomes reveals the genetics of pneumococcal meningitis. Nat. Commun., 10, 2176. 31092817 10.1038/s41467-019-09976-3 PMC6520353

[evo14590-bib-0036] McHenry, M.L. , Bartlett, J. , Igo, R.P. , Wampande, E.M. , Benchek, P. , Mayanja‐Kizza, H. , *et al.* (2020) Interaction between host genes and *Mycobacterium tuberculosis* lineage can affect tuberculosis severity: evidence for coevolution? PLoS Genet., 16, e1008728. 32352966 10.1371/journal.pgen.1008728 PMC7217476

[evo14590-bib-0037] Murphy, K. & Weaver, C.T. (2017) Janeway's immunobiology. 9th ed. Garland Science, Lond.

[evo14590-bib-0038] Oksanen, J. , Guillaume Blanchet, F.F. , Friendly, M.M. , Kindt, R.R. , Legendre, P.P. , McGlinn, D.D. , *et al.* (2020) vegan: community ecology package. R package version 2.5‐7.

[evo14590-bib-0039] Råberg, L. (2012) Infection intensity and infectivity of the tick‐borne pathogen *Borrelia afzelii* . J. Evol. Biol., 25. 10.1111/j.1420-9101.2012.02515.x 22536945

[evo14590-bib-0040] Råberg, L. , Hagström, Å. , Andersson, M. , Bartkova, S. , Scherman, K. , Strandh, M. , *et al.* (2017) Evolution of antigenic diversity in the tick‐transmitted bacterium *Borrelia afzelii*: a role for host specialization? J. Evol. Biol., 30, 1034–1041. 28345277 10.1111/jeb.13075

[evo14590-bib-0041] Radwan, J. , Babik, W. , Kaufman, J. , Lenz, T.L. & Winternitz, J. (2020) Advances in the evolutionary understanding of MHC polymorphism. Trends Genet., 36, 298–311. 32044115 10.1016/j.tig.2020.01.008

[evo14590-bib-0042] Razali, H. , O'Connor, E. , Drews, A. , Burke, T. & Westerdahl, H. (2017) A quantitative and qualitative comparison of illumina MiSeq and 454 amplicon sequencing for genotyping the highly polymorphic major histocompatibility complex (MHC) in a non‐model species. BMC Res. Notes, 10, 346. 28754172 10.1186/s13104-017-2654-1 PMC5534077

[evo14590-bib-0043] R Core Team . (2021) R: a language and environment for statistical computing. R Foundation for Statistical Computing, Vienna.

[evo14590-bib-0044] Salvaudon, L. , Héraudet, V. & Shykoff, J.a. (2007) Genotype‐specific interactions and the trade‐off between host and parasite fitness. BMC Evol. Biol., 7, 189. 17919316 10.1186/1471-2148-7-189 PMC2148064

[evo14590-bib-0045] Scherman, K. (2015) MHC polymorphism and host‐pathogen interactions. PhD thesis. Lund University, Lund, Sweden.

[evo14590-bib-0046] Scherman, K. , Råberg, L. & Westerdahl, H. (2014) Positive selection on MHC class II DRB and DQB genes in the bank vole (*Myodes glareolus*). J. Mol. Evol., 78, 293‐305. 24748547 10.1007/s00239-014-9618-z

[evo14590-bib-0047] ———. (2021) Borrelia infection in bank voles myodes glareolus is associated with specific DQB haplotypes which affect allelic divergence within individuals. Front. Immunol., 12, 703025. 34381454 10.3389/fimmu.2021.703025 PMC8350566

[evo14590-bib-0048] Spurgin, L.G. & Richardson, D.S. (2010) How pathogens drive genetic diversity: MHC, mechanisms and misunderstandings. Proc. R. Soc. B Biol. Sci, 277, 979–988. 10.1098/rspb.2009.2084 PMC2842774 20071384

[evo14590-bib-0049] Stoffel, M.A. , Nakagawa, S. & Schielzeth, H. (2017) rptR: repeatability estimation and variance decomposition by generalized linear mixed‐effects models. Methods Ecol. Evol, 8, 1639–1644.

[evo14590-bib-0050] Strandh, M. & Råberg, L. (2015) Within‐host competition between *Borrelia afzelii* ospC strains in wild hosts as revealed by massively parallel amplicon sequencing. Philos. Trans. R. Soc. London. B, 370, 20140293. 26150659 10.1098/rstb.2014.0293 PMC4528491

[evo14590-bib-0051] Tschirren, B. , Andersson, M. , Scherman, K. , Westerdahl, H. , Mittl, P.R.E. & Råberg, L. (2013) Polymorphisms at the innate immune receptor TLR2 are associated with *Borrelia* infection in a wild rodent population. Proc. R. Soc. B Biol. Sci, 280, 20130364. 10.1098/rspb.2013.0364 PMC3619520 23554395

[evo14590-bib-0052] Warton, D.I. , Wright, S.T. & Wang, Y. (2012) Distance‐based multivariate analyses confound location and dispersion effects. Methods Ecol. Evol., 3, 89–101.

[evo14590-bib-0053] Wei, T. & Simko, V. (2021) R package “corrplot”: visualization of a correlation matrix (Version 0.90).

[evo14590-bib-0054] Wickham, H. (2016) ggplot2: elegant graphics for data analysis. Springer‐Verlag, New York.

[evo14590-bib-0055] Wilson, D.E. , Lacher, T.E. & Mittermeier, R.A. (2017) Handbook of the mammals of the world: rodents II. Lynx Edicions, Barcelona, Spain.

[evo14590-bib-0056] Woolhouse, M.E.J. , Webster, J.P. , Domingo, E. , Charlesworth, B. & Levin, B.R. (2002) Biological and biomedical implications of the co‐evolution of pathogens and their hosts. Nat. Genet., 32, 569–577. 12457190 10.1038/ng1202-569

