# Supplementary material for: MHC class II genotype‐by‐pathogen genotype interaction for infection prevalence in a natural rodent‐Borrelia system
Source: Evolution. 2022 Aug 9;76(9):2067–75. doi: 10.1111/evo.14590 (PMC9541904; doi:10.1111/evo.14590)
Supplement: Supplementary file 1 — Fig S1. Frequency distribution of number of ospC strains per infected vole. Fig S2. Longitudinal data on number of ospC strains of infected voles. N = 48 voles, with two to four data points per vole. Lines are slightly “dodged” both x‐wise and y‐wise to reduce overlap. Fig S3. a) Proportion of bank voles carrying each DQB allele observed in the study population. Note that proportions given here are conservative estimates of allele frequencies in the population, as some individuals may be homozygous. b) Correlations among DQB alleles that occurred in at least 10% of bank voles. Alleles are ordered according to their frequency (decreasing from left to right). [file EVO-76-2067-s001.pdf]

Supplementary information: MHC class II genotype-by-pathogen genotype interaction for infection prevalence in a natural rodent-*Borrelia* system

Lars Råberg, Dagmar Clough, Åsa Hagström, Kristin Scherman, Martin Andersson, Anna Drews, Maria Strandh, Barbara Tschirren, Helena Westerdahl

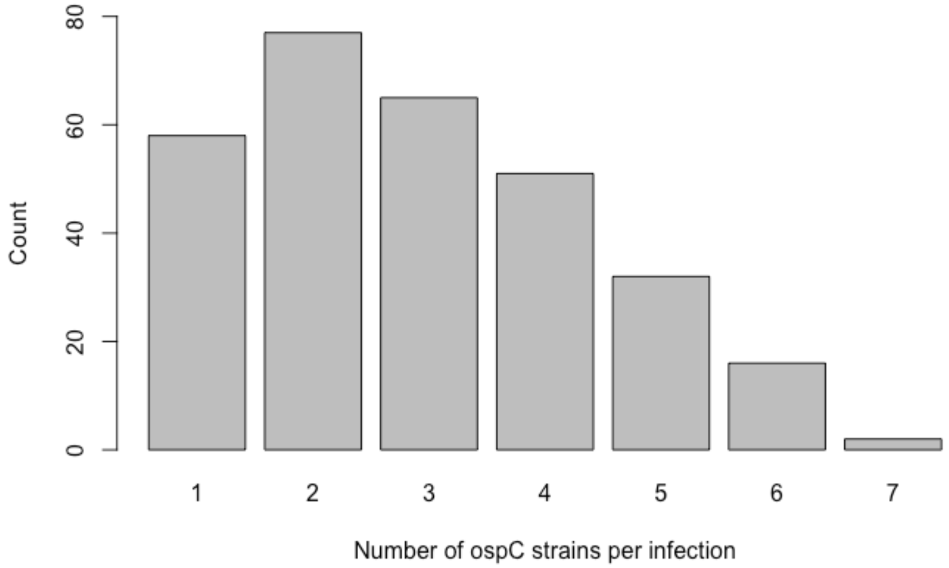

Fig S1. Frequency distribution of number of *ospC* strains per infected vole.

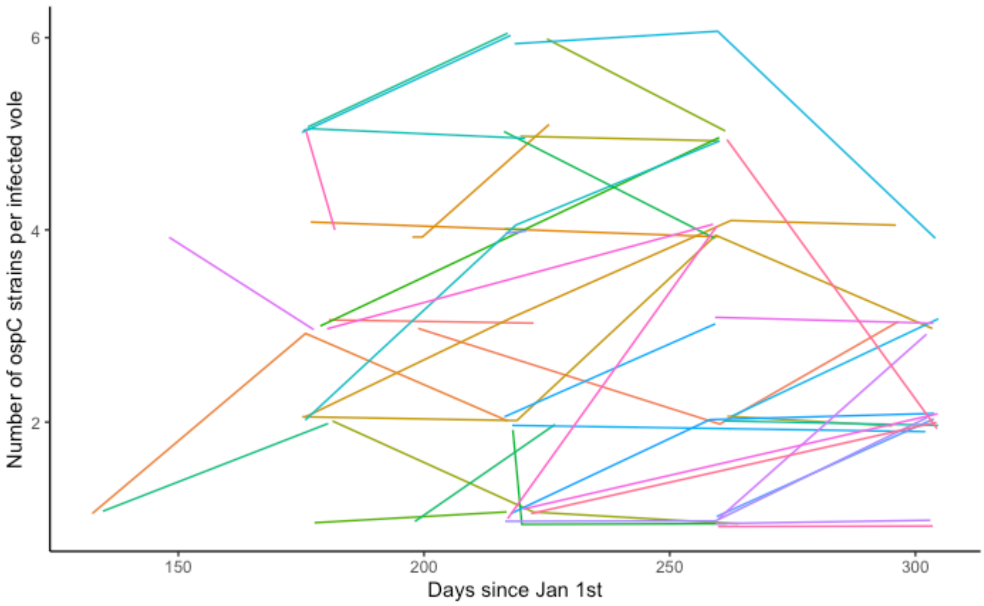

Fig S2. Longitudinal data on number of *ospC* strains of infected voles. N=48 voles, with two to four data points per vole. Lines are slightly “dodged” both x-wise and y-wise to reduce overlap.

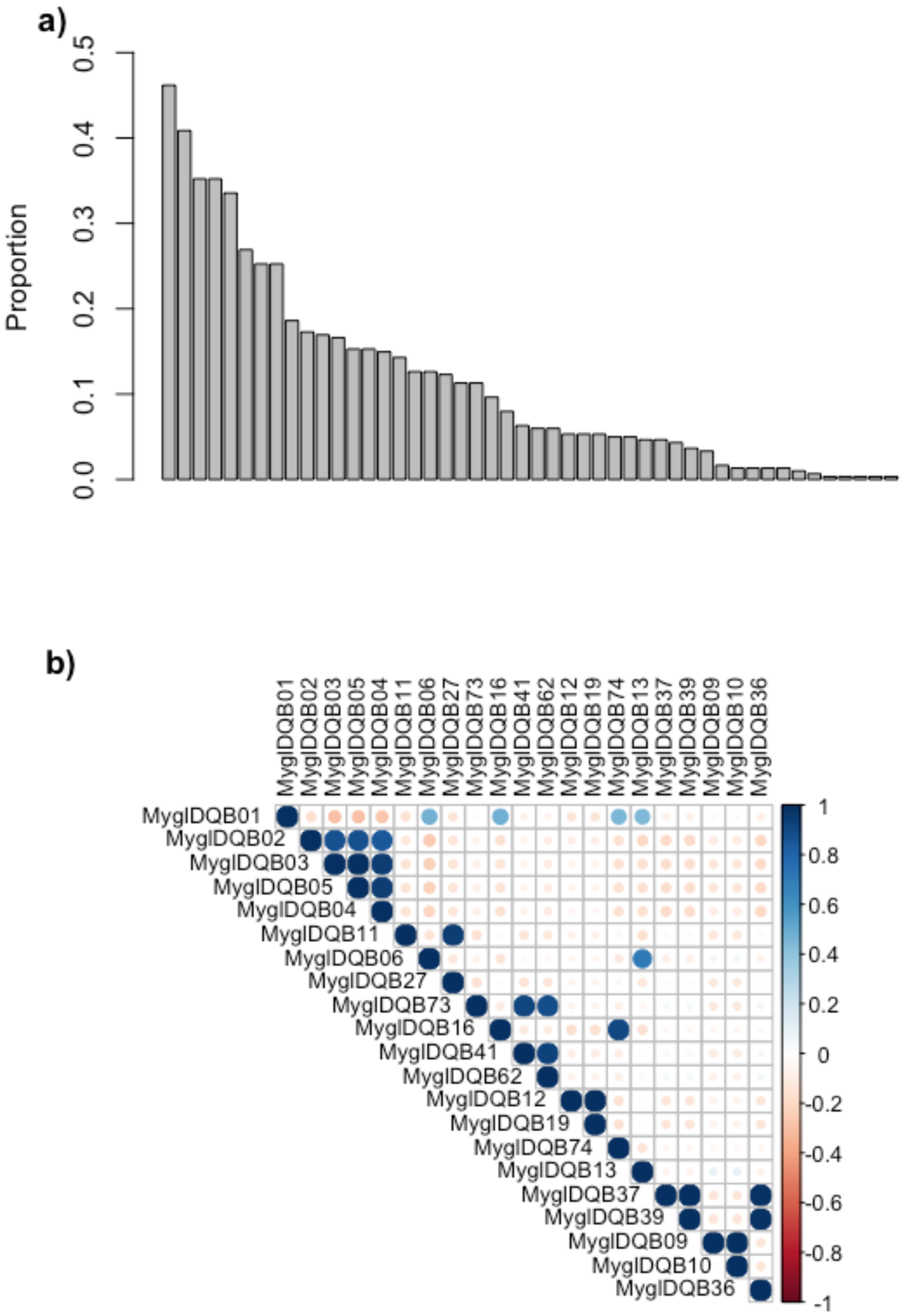

Fig S3. a) Proportion of bank voles carrying each *DQB* allele observed in the study population. Note that proportions given here are conservative estimates of allele frequencies in the population, as some individuals may be homozygous. b) Correlations among *DQB* alleles that occurred in at least 10% of bank voles. Alleles are ordered according to their frequency (decreasing from left to right).

Table S1. GLMM of effects of *ospC* strain, presence/absence of the MyglDQB*37 allele, and their interaction on prevalence. χ^2/df=0.97.

Effect	df	χ^2	Variance±SE	LRT[1]	P
ospC	6, 1978	79.29			<0.0001
MyglDQB*37	1, 1978	1.40			0.24
ospC× MyglDQB*37	6, 1978	13.17			0.040
Individual	1		0.102±0.059	6.85	0.009

[1]LRT=Likelihood Ratio Test statistic [χ^2=–2(log$L_{reduced\ model}$– log$L_{full\ model}$)].

Table S2. GLMM of effects of *ospC* strain, presence/absence of the MyglDQB*06 allele, and their interaction on prevalence. χ^2/df=0.97.

Effect	df	χ^2	Variance±SE	LRT[1]	P
ospC	6, 1978	77.9			<0.0001
MyglDQB*37	1, 1978	7.74			0.0054
ospC× MyglDQB*37	6, 1978	6.03			0.42
Individual	1		0.081±0.053	4.15	0.04

[1]LRT=Likelihood Ratio Test statistic [χ^2=–2(log$L_{reduced\ model}$– log$L_{full\ model}$)].
